# The Use of Mobile Games in the Management of Patients With Attention Deficit Hyperactive Disorder: A Scoping Review

**DOI:** 10.3389/fpsyt.2022.792402

**Published:** 2022-03-04

**Authors:** Haowen Jiang, Rohit Natarajan, Yao Kang Shuy, Lim Rong, Melvyn Weibin Zhang, Ranganath Vallabhajosyula

**Affiliations:** ^1^Lee Kong Chian School of Medicine, Nanyang Technological University, Singapore, Singapore; ^2^Family Medicine and Primary Care, Lee Kong Chain School of Medicine, Nanyang Technological University, Singapore, Singapore; ^3^Anatomy, Lee Kong Chian School of Medicine, Nanyang Technological University, Singapore, Singapore

**Keywords:** attention deficit hyperactivity disorder, children, serious games, digital technologies, systematic review

## Abstract

Attention deficit hyperactivity disorder (ADHD) is a common neurodevelopmental disorder associated with significant morbidity. Current treatment approaches consist of a mixture of pharmacological and psychological approaches. The emergence of digital technology, and mobile gaming applications, represents a promising novel method in potentially augmenting existing interventions for ADHD. In this review, we will map out the use of mobile gaming applications in the management of ADHD and evaluate the effectiveness of these technologies and any areas for future research. Four electronic databases were searched for relevant articles. All articles were screened for abstract and full text by two independent reviewers, and data extracted onto a common data extraction sheet. The data was narratively synthesized and reported in line with the PRISMA-ScR guidelines. A total of 19 studies were included. Studies mostly evaluated the effectiveness of games on male children with ADHD. Most games were focused on the treatment of ADHD, while a minority were focused on the diagnosis and monitoring of ADHD. Some of the common gaming mechanisms employed in games included having participants responding to cures, remembering details, and making associations between different entities. The studies generally showed an improvement in performance of children as they played the games, but evidence for the effectiveness of these modalities remains scarce and mixed. While it is exciting that there is such a wide variety of games available currently in the diagnosis, treatment, and monitoring of ADHD, many of the games lack clinical evidence to prove their effectiveness. Furthermore, most studies contain several limitations including small sample size, limited ages of participants, lack of control group, and lack of comprehensive outcomes. To promote the application of these games to clinical practice, robust clinical trials, collaboration between stakeholders and using a comprehensive set of outcome measurements is essential.

## Introduction

Attention deficit hyperactivity disorder (ADHD) is a neurodevelopmental disorder with a global prevalence of 5% in children aged 4–17 years old (1). It is classically characterized by symptoms of inattention, impulsivity, and hyperactivity. However, the clinical presentation of ADHD is often heterogeneous with many different phenotypes (1). It usually develops in early childhood, but diagnosis is most often established during schooling ages. Children who develop the disorder often have problems with education, social interactions, and often have other co-existing mental illnesses (2).

Conventional treatment strategies for ADHD include a combination of pharmacological and psychological approaches, based on the National Institute for Health and Care Excellence (NICE) guidelines (3). However, pharmacological treatment is not effective in up to 36% of patients, with multiple side effects (4). Furthermore, adherence to medications is also a problem in children with ADHD (5). To address these issues, digital therapeutics has presented itself as a possible solution. Among digital therapeutics, one promising tool is serious gaming or gamification (6). Computer games are hugely popular, being played by millions of adolescents and adults around the world. Furthermore, high-quality computer games have been shown to improve cognitive functioning, including memory, concentration, and behaviors (6). These games are most often displayed on a personal computer (PC) or a desktop but can also be displayed on other modalities such as mobile phones, tablets, video console, or 3D device (7).

Video games have been previously shown in reviews to be effective in both the diagnosis and treatment of ADHD and other mental health disorders (7, 8). One of the proposed mechanisms as to how video games are so effective is *via* “gamification.” It is proposed that the rewarding feature of games is more attractive to children and thus improves adherence (7). Video games have also been shown to increase motivation, cognition, emotional wellbeing and social interactions (9), which are all features welcome in patients with ADHD. However, a cross-sectional study by Schou Andreassen et al. has also found a positive correlation between addictive use of video games and increased symptoms of ADHD (10). It appears that video games have the potential to be beneficial but may cause increased harm if used excessively. Furthermore, as inattention is a core feature of ADHD, long-term engagement of children with ADHD may be difficult (7). There is thus a need to map out the use of gaming applications in ADHD and identify if such applications provide a net benefit. While these games have mainly been used in the treatment of ADHD, they also have potential to be used in other aspects of the management of ADHD, including diagnosis, education, monitoring.

The recent technological progress has led to the development of powerful and portable devices and led to the emergence of mobile health (mHealth). Five broad classifications of mHealth include health applications, smartphone-connected devices, wearable and wireless devices, handheld-imaging platforms, and miniaturized sensor-based technologies (11). In this review, we will be focusing only on one aspect of mHealth which is the use of health applications. The use of mHealth technologies has been shown to promote self-management in non-communicable diseases (NCDs), especially in young people (12). Combined with the potential of benefits of video games, this has potential to have a large impact in the management of ADHD.

Peñuelas-Calvo et al. (7) have previously performed a systematic review of video games both for the assessment and treatment of ADHD (7). While their review was comprehensive and one of the first in the field to map out the existing evidence in the literature for games for ADHD, their review had several limitations. The authors have included video games that served either as an assessment or intervention tool, and not reviewed games that could be psychoeducational in nature, or games that are meant for other issues relating to ADHD. Their review was also limited to studies that have reported outcome data. Given the rapid advances in technologies, and how rapidly games are being developed, it makes sense to broadly scope out all the ADHD games, instead of sticking to the limitations as set forth by Peñuelas-Calvo et al. (7).

Thus, the aim of this review is to map out all existing video games for ADHD, and in determining the specific skill sets that each game targets and their outcomes. It is also the aim of this review in examining the gamification techniques or serious game strategy that has been adopted in existing published ADHD games.

The findings arised from this study would have resultant research and clinical implications. In terms of research, this will help researchers understand the effectiveness of ADHD games, and techniques that might help these games be effective. From a clinical perspective, this would help provide guidance for clinicians attempting to use games as a modality of treatment intervention.

## Methods

This scoping review was reported using Preferred Items for Systematic Reviews and Meta-analysis extension for scoping review (PRISMA-ScR) guidelines (13).

The bibliographic databases of Cochrane, MEDLINE, PsycInfo and Scopus were searched for all records published within the past 11 years to identify potentially relevant articles. To identify the relevant articles, the following keywords were used for the search: “Play and Playthings,” “Games,” “Recreational,” “Video Games,” “Cell Phone Use,” “Telemedicine,” “Telerehabilitation,” “Mobile Applications,” “Computers,” “Handheld,” “Attention Deficit Disorder with Hyperactivity.”

The details on the search strategy can be found in Supplementary Appendix 1. The search strategies were drafted and refined through team discussion amongst the authors and the librarian from Lee Kong Chian School of Medicine. The keywords were adapted for the different databases. The final search results were exported into EndNote and the duplicate records were removed. These searches were supplemented by hand searches of the references listed in the included articles. This was an iterative process, repeated until no new articles were identified. The search was conducted in July 2021.

Articles were included if they were (a) articles on digital games that are used on a mobile or tablet platform, with or without another digital modality, (b) targeted patients with ADHD or caregivers of patients with ADHD, (c) a form of primary research, (d) peer-reviewed articles, pre-prints, or gray literature. Articles were excluded if they were (a) published in a language that is not in English, (b) published before 2010, (c) systematic reviews or other forms of secondary research.

The articles were independently screened by two reviewers (RL and RN) using Covidence, who then extracted and categorized data from the selected articles into a spreadsheet. The reviewers sequentially evaluated the titles, abstracts, and then full texts of all publications identified by the searches for potentially relevant articles. If there were any disagreements between the reviewers, this was discussed with a senior study team member.

The data extracted from each of the article includes publication details (e.g., title, author, publication date), conduct of the study (study design, institution), and article content (description of games, research question and answer, important discussion points). The data was extracted by 4 reviewers (RL, RN, JHW, SYK) onto a common data extraction sheet on Microsoft Excel. The data was then synthesized into figures and tables and described according to the PRISMA-ScR guidelines.

An additional search for games intended for use among children with ADHD was also conducted. The keywords “ADHD” and “game” were entered into the Google Play Store and Apple Store. Games were included if the description of the game included ADHD. General information and features of the highlighted games were used to supplement the data collected from articles in this scoping review to provide enhanced insight on the types of games commercially available on the market.

## Results

**Figure 1:**
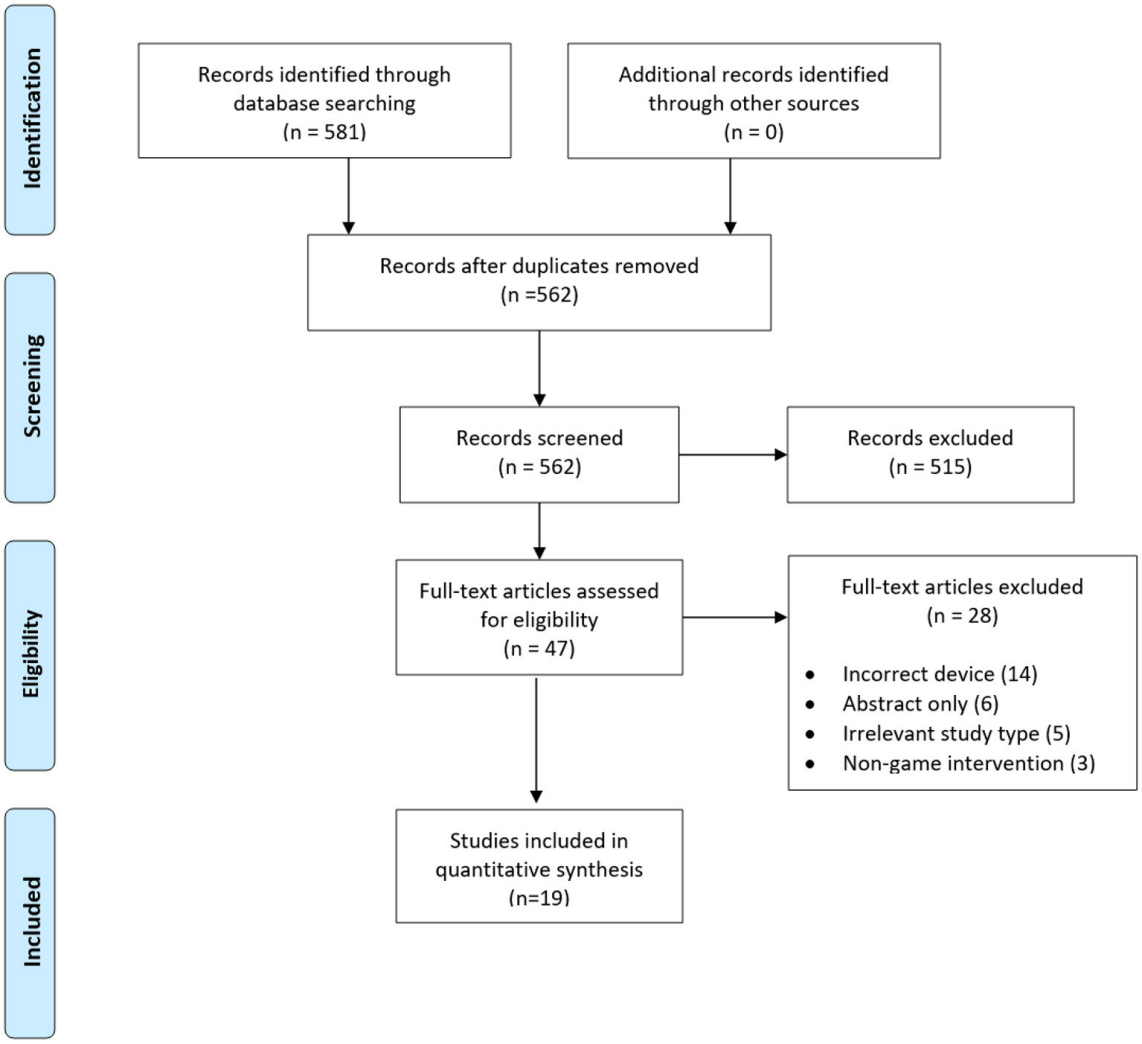
PRISMA flowchart of the study selection process.

The overview of the selection of the studies are summarized in Figure 1. A total of 581 citations were identified after the initial search strategy, of which 17 were duplicates. Five hundred fifteen studies were further excluded after title and abstract screening. Forty-seven full-text articles were assessed for their eligibility in this review with 19 studies meeting the inclusion criteria.

The general characteristics such as the study design, demographic characteristics, and study sample of various studies are summarized in Table 1.

**Table 1:** General characteristics of reviewed studies.

**S/n**	**Study**	**Design**	**Country**	**Sample size**	**Mean age (age-range)**	**% Male^a^**
1	Agustini et al. (14)	Mixed methods^b^	Indonesia	19	(7-8)^c^	Not reported
2	Baghaei et al. (15)	Mixed methods^b^	New Zealand	36	(20-29)^c^	47.2
3	Craven and Groom (16)	Developmental	UK	–	–	–
4	De La Guía et al. (17)	Mixed methods^b^	Spain	12	7.83 (5-16)	66.7
5	De La Guía et al. (18)	Developmental	Spain	–	–	–
6	Fraiwan et al. (19)	Developmental	Jordan	–	–	–
7	Harrison et al. (20)	Mixed methods^b^	USA	4	11.75 (11-12)	75
8	Ivett Daniela Jácome et al. (21)	Mixed methods^b^	Colombia	5	Not reported	60
9	Mwamba et al. (22)	Mixed methods^b^	South Africa	30	10 (5-16)	53.3
10	Ocay et al. (23)	Experimental (ethnographic)	Philippines	11	(6-10)^c^	83.3
11	Rodríguez-Pérez et al. (24)	Developmental	Spain	–	–	–
12	Sinnari et al. (25)	Experimental (ethnographic)	UK	17	(6-12)^c^	0
13	Somma et al. (26)	Developmental	Italy	–	–	–
14	Kollins et al. (27)	Experimental (open-label, non-randomized)	USA	206	10.6 (8-14)	74.8
15	Spitale et al. (28)	Mixed methods^b^	Italy	27	26 (19-44)	55.6
16	Swarts et al. (29)	Developmental	South Africa	–	–	–
17	Tajima-Pozo et al. (30)	Case report	Spain	1	10 (10)	100
18	Wronska et al. (31)	Mixed methods^b^	Poland	6	(8-12)^c^	33.3
19	Yerys et al. (32)	Experimental (before-and-after)	USA	19	11.25 (9-13)	89.5

*UK, United Kingdom; USA, United States of America*.

a *Percentage male participants*.

b *Combination of developmental and some experimental methods*.

c *Mean age not reported*.

Developmental research is defined as the systematic study of designing, developing, and evaluating instructional programs, processes, and products that must meet criteria of internal consistency and effectiveness (33). These studies mainly focus on outlining the information and theories used in the development of mobile games.

Of the 19 studies included in this review, 6 studies (32%) were purely development research papers with no measured outcomes (16, 18, 19, 24, 29, 34), 4 studies (21%) were experimental designs (23, 25, 27, 32), 8 studies (42%) were mixed methods studies (developmental studies which measured some primary outcome) (14, 15, 17, 20–22, 28, 31), and 1 paper (5%) was a case report (30).

Among the 4 experimental studies, 1 study was an open-label, non-randomized trial (27), 2 were ethnographic studies (allowing participants to use the application while data was collected from the interaction) (23, 25), and 1 study was a before-and-after study [measuring outcomes before and after intervention (32)]. There are 4 mixed methods studies utilized surveys to gather primary data (21, 22, 28, 31), 2 studies utilized a before-and-after experimental design (14, 17), and 1 study used a withdrawal study design (20). Notably, none of the studies included were randomized controlled trials (RCTs).

The studies were conducted in a wide variety of different countries, with most studies being conducted in Spain (4 studies; 21%) or the USA (3 studies; 16%). Four studies (21%) were conducted in low-income countries with the rest being conducted in high-income countries.

Excluding the case report, 12 studies (67%) involved ADHD patients with or without other neurodevelopmental disorders such as autism spectrum disorder (ASD) or involved caregivers in the study design. Some studies were primarily developmental but included participants for surveys. The median number of participants was 18. Most studies involved the use of groups of children <16 years old, with only 2 studies (11%) involving the use of participants above the age of 16. Most studies also involved mostly male participants, with only 3 studies (16%) involving more females than males.

The games modalities that were observed in the review are summarized in Table 2. Based on our review, we analyzed a total of 18 games. Of these 18 games, 15 games were primarily designed to treat children with ADHD or symptoms of ADHD, 2 games were designed to assess the presence of ADHD in children, while one game was used to monitor the symptoms of ADHD in children. Note that while the games were sorted based on their primary objectives, some games had overlaps in functions. For example, the games described by Craven et al., was primarily designed to monitor symptoms of ADHD (16), but could potentially be a therapeutic tool for ADHD in children.

**Table 2:** Game modalities.

**S/n**	**Study**	**Name**	**Acquisition**	**Description**	**Aim of game**
1	Agustini et al. (14)	Bible warriors adventure games	Developed	Unavailable	Intervention (Facilitate learning and education in children with ADHD)
2	Baghaei et al. (15)	GLtron (modified)	Commercially acquired (modified)	Modification of the GLtron video game to include BRIGHT, a set of generic design principles aimed to increase self-esteem in ADHD children because of playing computer games	Intervention (improving self-esteem in children with ADHD)
3	Craven and Groom (16)	SnappyApp, awkward owls, wormy fruit	Developed	All three games are based on Go/No Go tests—Fruits are flashed one after another, players must click on “bananas” when it appears (SnappyApp); Players are tasked to collect only yellow owls and ignore brown owls (Awkward Owls); Players click Go on “non-wormy” fruits, stop when a “wormy apple” appears (Wormy fruits)	Monitoring of ADHD symptoms; potential for training of inhibitory control
4	De La Guía et al. (17)	Stimulating capabilities (StiCap)	Developed	StiCap comprises three games that aim to enhance memory (a memory card game), attention (memorizing order of animals that appear on screen), and associative capacity (selecting animal whose name starts with letter flashed on screen). Each game has 3 difficulty levels that are activated whenever a previous level of lower difficulty is cleared	Intervention (Improving cognitive abilities e.g., memory and attention in children with ADHD)
5	De La Guía et al. (18)	Stimulating collaborative cognitive capabilities (Co-StiCap)	Developed	Players are given a series of pictures attached to NFC tags. The game will project pictures on a screen, and players must tap the NFC tag of the corresponding picture on an NFC reader	Intervention (Facilitate stimulation of cognition and communication in children with ADHD)
6	Fraiwan et al. (19)	Unnamed game 1 (S/n 6)	Developed	There are four game components, two of which require action by players—(i) Behavior game (Players decide what to do in certain social situations to gain rewards), (ii) Puzzle and matching game (Involves jigsaws and memory games)	Intervention (Acquisition of appropriate behavioral patterns in children with behavioral disabilities)
7	Harrison et al. (20)	EpicWin	Commercially acquired (but modified)	Game tasks players to complete a to-do list—where completing one item grants your character XP and allows you to complete quests	Intervention (Promotion of self-regulation in attention and task completion in children with ADHD)
8	Ivett Daniela Jácome et al. (21)	DIVIDI2	Developed	Players turn into an astronaut and have access to different kinds of adventures which test different concepts e.g., gamification theory, usability, design patterns, and EMOINAD cards. Players can also create a virtual pet which can be customized according to players' performance in the game	Intervention (Improve divided attention i.e., skill to pay attention to more than one stimulus without losing focus on current task in children with ADHD)
9^*^	Mwamba et al. (22)	PANDAS^*^	Developed	The objective of the task was to travel on a raft from one end of a river to the other end as quickly as possible, while collecting gems and avoiding obstacles by shifting the raft to one of three lanes	Assessment (Assess presence of ADHD using quantitative methods)
10	Ocay et al. (23)	Unnamed game 2 (S/n 10)	Commercially acquired on Google Play Store	Players are exposed to “find-the-object” game play	Intervention (Improve learning motivations and frustration tolerance in children with ADHD)
11	Rodríguez-Pérez et al. (24)	mHealth tool (generic name)	Developed	This application acts as a hub that integrates four platforms for stakeholders in the management of ADHD in children: children, parents, teachers, and medical staff. The children's platform has three different serious games—color sets, card games, and mathematical games	Assessment (using expert systems based on Bayesian networks); Intervention (Development of cognitive abilities of children with ADHD)
12	Sinnari et al. (25)	ACTIVATE	Commercially acquired	ACTIVATE™ comprises three portals—the teacher, student, and test portal. The student portal comprises six computer games which target a different set of skills—from category formation and sustained attention to speed of information processing. The test portal involves the player taking three NIH cognitive assessments (The Flanker Test, The Working Memory Test, The Go/No-Go Test)	Intervention (Improve attention, processing, and cognitive capacities of children with ADHD)
13	Somma et al. (26)	Unnamed game 3	Developed	The game has 2 phases—assessment and training. The testing section evaluates a child's degree of ADHD based on four fields—attention, working memory, inhibition, and planning. The training area provides specific training, with respect to each area presented in the assessment phase, through exercises and tasks that adapt to the child's starting level	Intervention (Improve executive functions in children with ADHD)
14	Kollins et al. (27)	AKL-T01 (EndeavourRx)	Commercially acquired	Players are required to navigate an avatar through obstacle courses and collect targets along the way. Clearing each level requires a certain degree of focus and multi-tasking ability. Levels adapt to the competency level of children	Intervention (Improve attention)
15	Spitale et al. (28)	Reflex	Developed	Players are required to place the physical item they see on the application on the physical play area in front of them. There are five activities of five tasks in total—images, numbers, letters, words, tangram, with each task lasting a maximum of 2.5 min	Intervention (Improving motivation and lowering frustration in people with neurodevelopmental disorders)
16	Tajima-Pozo et al. (30)	ADHD trainer	Commercially acquired (Play Store/App Store)	The game is based on the Tajima cognitive method (TCT), with activities targeting different cognitive areas including calculation, verbal fluency, attention, perceptual reasoning, visuomotor coordination, and memory	Intervention (Improve executive function and cognitive symptoms in children with ADHD)
17	Wronska et al. (31)	LyC: Lectura y Comprensión	Developed	The game had nine interactive exercises which proceeded in three stages. Students had to (i) read text containing important information, (ii) understand and process information into answers, and (iii) select the correct answer from all presented by dragging the button from the toolbox and dropping it on the picture or label	Intervention (Improve comprehension and attention of children with ADHD)
18	Yerys et al. (32)	Project EVO	Commercially acquired (Google Play Store)	There are two main interventions in the application—multi-tasking and educational interventions. In the multi-tasking intervention, players rapidly switch between a perceptual discrimination attention/memory task (choosing a target animal from three options) and a continuous visuomotor driving-type task (steering a hovercraft down a river). In educational intervention, players generate words from an array of letters	Intervention (Target cognitive interference in children with ASD and co-occuring ADHD symptoms)

*Developed, Developed by the research team specifically for the purposes of the study*.

*Commercially Acquired, Acquired from openly available sources including Google Play Store, Apple App Store, or other available sources for mobile games (unspecified in papers)*.

* *PANDAS was described in both Mwamba et al. (22) and Swarts et al. (29). Therefore, the description for PANDAS was collapsed under one row*.

As to where the 18 games were acquired from, 11 games were developed by the research teams, while 7 games were commercially acquired, through the Google Play Store, Apple App Store, or other available sources for mobile games.

The 17 games analyzed can be grouped into broad categories, based on the features of their gameplay. Note that there was one game with no game description and some games can fall under more than one category.

There were 6 games (15, 16, 22, 23, 25, 27, 30, 34) in which the players responded to specific cues, execute specific actions according to instructions or to stimuli displayed on the application. For example, SnappyApp (16) required players to select only the bananas when they appear. In other games, the players have to avoid the obstacles (22), find the objects (23) or follow the instructions (25).

There were 4 games (17, 19, 30, 34) that involved a memory component as the core of the game. In these games, the players must remember a particular order or sequence of items, and accurately recollect them after a period. For example, in the game Sti-Cap (17), players are required to memorize the order of animals that appear on screen, and play a memory card game.

In 7 games (17, 18, 28, 31, 32, 34, 35) the players were required to make an association between two entities. For example, in the game Reflex (28), players are required to match the item shown on screen with the physical item on a scanning field.

Other, smaller categories include behavioral games (19), which challenges players to choose options that are socially appropriate for a given setting; rewards for tasks (20); and adventure games (21).

A total of 13 studies were experimental or developmental studies that involved the collection of quantitative and qualitative data from a sample population for further analysis. The observations derived from the studies were summarized in Table 3. There were 12 studies that were targeted at treatment of patients with ADHD, while only one study collected information that aids in the screening of potential patients with ADHD (22).

**Table 3:** The measured outcome and the results that employed gamified interventions.

**S/n**	**Study**	**Name of game**	**Measured outcome**	**Outcome result**
1	Agustini et al. (14)	Bible warrior adventure games	Field trial test (pre/post)	Average score increased from 67.01 to 80.04
2	Baghaei et al. (15)	GLtron (modified)	Subjective quantitative and qualitative evaluation by participants	Game increases confidence in children with low self-esteem (81%) Game keeps players motivated (79%) Will spend more time playing modified version compared to original (76%)
3	De La Guía et al. (17)	Co-StiCap	Smileyometer test (satisfaction) Task performance and interactions (effectiveness)	Games were brilliant (*n* = 7), really good (*n* = 3), and good (*n* = 2). No negative inputs Increase in the number of successes per children with the system (*p* = 0.005) Decreased task performance time with the system (*p* = 0.001) Child performed more physical and verbal interactions with the system (*p* < 0.001)
4	Harrison et al. (20)	EpicWin	Reading engagement, response and completion Acceptability	Improvement in reading engagement with small effect size (*p* < 0.01) Improvement in reading response engagement, reading response completion and reading response accuracy with moderate effect size (*p* < 0.01 for all) Game was an acceptable strategy (*m* = 5.54 out of 7)
5	Ivett Daniela Jácome et al. (21)	DIVIDI2	Simultaneous interview and questionnaire with children and parents to measure usability and acceptance	Children will play with it (100%) and with their friends (80%) Parents consider that children will accept game (100%)
6	Kollins et al. (27)	EndeavorRx	ADHD-related impairment measured by impairment rating scale (IRS), clinical global impressions scale—improvement (CGI-I) and ADHD rating scale (ADHD-RS) Safety result	Significantly improved IRS, CGI-I and ADHD-RS with or without stimulant use after 4-week treatment and 4-week treatment pause (*p* < 0.001 for all) 18% of participants experienced a mild or moderate device-related adverse event (decreased frustration tolerance, headache, and irritability)
7	Mwamba et al. (22)	PANDAS	Sensitivity and specificity of linear binary support vector machine (SVM)	Calculated accuracy of 83.5% Sensitivity of 75% on test set Specificity of 100% on test set Test set *n* = 7
8	Ocay et al. (23)	Unamed	Time spent using augmented reality (AR) compared to time spent using mirror tracing persistence task (MTPT) as indicator of frustration	Increase in task attempt time using AR (*p* = 000)
9	Sinnari et al. (25)	ACTIVATE	Sustained attention, working memory, speed of information processing, response inhibition, cognitive flexibility, category formation, pattern formation, and multiple simultaneous attention National institute of health (NIH) assessments	Improvement in sustained attention (23%), response inhibition (28%), speed of processing (49%), and cognitive flexibility (38%) For NIH assessments, enhancement in flanker test (20%), Go/No Go test (24%), and working memory test (80%)
10	Spitale et al. (28)	Reflex	Interviews with therapists (descriptive feedback) Game performance scores (effectiveness)	Minimalism, predictability, clarity, reward, configurability, levelability, assessability, constrainability, avatar were themes highlighted by therapists Activities that require active manipulation of object activated by a digital simulation with real time feedback were most suitable (Images, Letters, and Tangram)
11	Tajima-Pozo et al. (30)	ADHD trainer	Conners parent and teacher rating scales (CPTRS) and Barkley school situations questionnaire (BSSQ)	Improvement in CPTRS (19–15 for teachers, 20–16 for parents) Improvement in BSSQ (main severity score decreased from 70 to 66)
12	Wronska et al. (31)	LyC: Lectura y Comprensión	Mean score and time taken to complete game System usability scale (SUS)	Increased mean score (2.83–3.17) Decreased mean time (86.17s−40.00s) SUS A grade (89.58 of 100)
13	Yerys et al. (32)	Project EVO	Test of variables of attention attention performance index (TOVA API) Descriptive caretaker reports	For multi-tasking intervention (MTI): No significant gain in TOVA API, but medium-to-large effect size Parents report significant reductions in ADHD-RS-IV, BRIEF-2, SSIS Problem behaviors with large effect No significant gains and small-to-medium effect size on the CANTAB's spatial working memory, and parent ratings on the SSIS Social Skills For Educational Intervention (EI): No significant gains or losses on the primary outcome of the API from the TOVA. However, this group showed an overall worsening on the API with a small-to-medium effect size Non-significant reductions in ADHD-RS-IV, BRIEF-2, and SSIS problem behavior scores, although these reductions were associated with medium-to-large effect sizes No significant gains and small-to-medium effects were found on the CANTAB spatial working memory and parent ratings on the SSIS social skills

In our analysis, studies were split into those that presented purely objective data (*n* = 4) (14, 23, 25, 27), purely subjective data (*n* = 3) (15, 21, 30), and those that presented both data types (*n* = 5) (17, 20, 28, 31, 32).

The participant performance over the period as they played the game were the most common objective data observed in four studies (*n* = 4) (17, 23, 28, 31). The objective data was based on the indicators such as game score, time taken to complete task being present in three of these studies (23, 28, 31). Special indicators such as time taken before participants give up (23) and the frequency of physical and verbal interactions with the system (17) were used as a benchmark for participant frustration tolerance and participant engagement, respectively.

There were studies that used a formal assessment tool to gauge the effectiveness of the gamified intervention (*n* = 3) (25, 27, 32), namely using National Institute of Health (NIH) assessments (25), Test of Variables of Attention Attention Performance Index (TOVA API) (32) as well as the Impairment Rating Scale (IRS) (27) and Clinical Global Impressions Scale—Improvement (CGI-I) (27) to evaluate the improvement of ADHD symptoms among participants of the study.

There were studies that used functional assessments as metrics of ADHD performance (*n* = 2) (14, 20), where Field Trial Tests (14), and reading or reading response task performance (20) were used to gauge the ability of children with ADHD to accomplish tasks that are expected of them in the real world.

All studies in this category demonstrated that the use of gamified interventions can prove to be helpful in patients with ADHD in at least one aspect except for Yerys et al. (32) which found no significant changes in TOVA API metrics. No study raised concerns of statistically significant worsening of ADHD symptoms after intervention, although publication bias was not accounted for in this scoping review.

The quantitative data on the acceptance of gamified interventions by parents, children, or both (*n* = 5) (15, 17, 20, 21, 31, 32) are group under the subjective data category. Results from these articles suggest that such games were well-accepted by both parents and children, who welcomed the idea of a novel modality of treatment for children with ADHD.

Quantitative data was also collected on the effectiveness of these treatments (*n* = 2), with the Conners Parent and Teacher Rating Scales (CPTRS) and Barkley School Situations Questionnaire (BSSQ) being used by one study (30), and the ADHD-RS-IV, BRIEF-2, SSIS Problem behaviors, CANTAB Spatial Working Memory and SSIS Social Skills being used by another (32).

Results obtained were mixed, with Tajima-Pozo et al. (30) suggesting improvements in the measured indicators, while Yerys et al. (32) exhibited improvements in only one category, and non-significant changes in others. Descriptive feedback on a game (*n* = 1) was also provided.

The themes analyzed by this paper should be considered by future developers who wish to create serious games for children with ADHD.

## Discussion

Serious games targeted to treat patients with ADHD, especially children, often rely on the use of colorful visuals to leverage the idea that children tend to be more visual learners, and that knowledge uptake and retention is often better using visuals as opposed to other modalities of learning (19).

The most common features in many of the serious games reviewed, were clear set of objectives, feedback upon the achieving the objectives, internal reward systems (21, 22) and positive affirmation (15). Inclusion of game design principles, definite objectives and positive affirmation indeed increase the self-esteem in ADHD children, motivate them to play the game (15). The feedback and the reward system in the game provides the children with sense of progress and help them to maintain the attention to a particular task. This feature is especially important in the context of ADHD, where children get distracted easily, which can be exacerbated when they do not receive signals that they are making progress (31).

Another essential feature that was present in the serious games analyzed was portability and location independence. Serious games promote self-management and allow patients to undertake the intervention without constraints on space or time (14, 17, 20). This would potentially limit the multiple clinical visits by the patients for the conventional therapies. This fact is further highlighted in the use of mobile devices, such as hand phones or tablets that are easy to carry around and easily accessible.

The games were developed to enhance the attention, memory, and behavioral characteristics. The attention-based games are based on Go/No-Go test concept. The Go/No-Go test essentially requires subjects to respond to a “Go” stimuli, and inhibit a response to a “No-Go” stimuli, and is often used to measure sustained attention and response control (36). For example, the game AKT-T01, better known as EndeavorRx, the first ADHD intervention game to receive FDA approval, required players to move their character away from obstacles and move toward targets on screen (37). The memory games aimed to improve the associate capacity and cognition among the ADHD children. There was limited evidence to extrapolate that the improvement of memory through serious games to the real world context (38).

The game genres were derived from the aims of each respective game. Games that primarily aimed to improve attention amongst subjects with ADHD employed features that required players to focus on a particular task, or execute a task based on a particular cue. Often the features of these games, directed at improving or assessing attention, are based on a Go/No-Go test concept. The Go/No-Go test essentially requires subjects to respond to a “Go” stimuli, and inhibit a response to a “No-Go” stimuli, and is often used to measure sustained attention and response control (36). For example, the game AKT-T01, better known as EndeavorRx, the first ADHD intervention game to receive FDA approval, required players to move their character away from obstacles and move toward targets on screen (37). The games developed to improve the behavioral aspects by training the players to select the most socially appropriate option in a given gamified social context (19), these games are also well-suited for other neurodevelopmental disorders. There is lack of validation of these games, hence the efficacy remains to be seen.

It is important to acknowledge the platforms in which the games have been deployed. As smartphone capabilities improve, the mobile platform is widely used as a range of applications that can be executed on them.

The Phygital approach is novel in mobile gaming, which bridges the digital world with real world and provides an extensive immersive experience. An example of a mobile game that utilizes this approach is the Reflex game (28). The game uses the device's camera that captures the user's interactions and reflect them in the application. These types of emerging technologies have opened the possibilities of treatment of ADHD using mobile games.

Another innovative modality in the treatment of ADHD with mobile games is the use of Augmented Reality (AR) and Virtual Reality (VR). For example, an AR-based mobile application was used to perform Augmented Reality simulations through interactive “find-the object” game play in one of the included study (21). The results showed improved frustration tolerance measured using the Mirror Tracing Persistent Task (MTPT) which is a standardized psychological test for quantifying frustration. This indicates that AR improves efficacy in the use of mobile games to treat ADHD. VR was implemented in “The Secret Trial of Moon” (39) to provide an interactive cognitive training environment through chess, while AR has been conceptualized to be implemented into games for treatment of children with ADHD (40). VR and AR allows for a more immersive gaming environment which may be advantageous in a therapeutic setting However, as this is a new and rapidly advancing field, more research is ultimately required.

The other common gaming platform reported in the literature were personal computer (PC) games. Plan-it-Commander (41), UVAMat (42), and ADDventurous Rhythmic Planet (43) are some examples of the PC games that are under development or validation. Though, the PC games share similarities with mobile games, they hold an edge owing to their ability for customization, allow cross platform integration, greater flexibility, and processing performance.

Sensor driven platforms, such as a Brain-Computer Interface (BCI), are increasingly being explored as a possible modality for serious games in the treatment of ADHD, with preliminary evidence suggesting that BCI can be effective at minimizing ADHD symptoms (44). These sensors rely on real time data to guide a visual PC interface. Park et al. developed a game leveraging the data from electroencephalograms (EEGs) to guide game elements (45).

The greater processing power of PC platforms allows for a greater diversity of performance data to be collected, allowing for an improved assessment of cognitive function and attention to be measured, providing more information to aid clinicians in the management of the child's symptoms.

However, a significant shortcoming of PC platforms is the lack of portability, which can significantly impair treatment accessibility and lead to poorer patient compliance should it be administered as part of a treatment plan. Additional hardware such as EEG equipment required to operate complex systems such as the BCI require centralized provision of care, negating the initial advantage of serious games as a treatment modality.

There were 2 studies (15, 27), that explored the immediate post-study effects of the gamified approaches, however the long-term impact of the approach on the improvement of ADHD symptoms were not studied. There were limited studies that intended to explore the long-term impact of the gaming interventions on treatment of ADHD Baghaei et al. (15), Kollins et al. (27).

Future studies attempting to elucidate an association between a gamified approach to the treatment of ADHD and effective management of ADHD can consider using validated tests and designing studies that allow the observation of the long-term impact of the approach.

Whilst the use of mobile games in the treatment of ADHD seems interesting, research hints that medication coupled with behavioral therapy may produce better outcomes in the management of ADHD symptoms (46). This seems to elucidate that combination therapy with multiple interventions is more effective than single intervention. Whilst the effectiveness of mobile games in the treatment of ADHD requires more research to confirm efficacy, it perhaps can be used as an adjunct treatment coupled with traditional medication and behavioral therapy to achieve better symptomatic management in ADHD patients. This subject, however, requires further detailed research to be confirmed.

Preliminary evidence suggests that game-based interventions are positively received by both patients and caregivers, likely a result of games being seen as less invasive than pharmacological therapy and a novel treatment modality. Most caregivers also report visible improvements in the child's ADHD symptoms after exposure to the game. Furthermore, given the likely lower side effect profile of game-based interventions, caregivers are more willing to opt for such modalities given that it is simple to prescribe as well as discontinue use if necessary.

## Limitations of Included Studies

There are some limitations of the studies analyzed in this scoping review, some of which significantly impacted the strength of the evidence produced by the studies, resulting in research gaps that cannot be adequately answered.

### Study Design

Several flaws in study design were identified. Most studies involved very small sample sizes (median participant size = 18). Only 1 study (27) exceeded the recommended minimal sample size of 50 for experimental studies (47). While this number is adequate to test 90% of usability problems (48), it is insufficient to determine the treatment efficacy of such games. Four studies attempted to quantify efficacy using improvements in game scores, without accounting for the natural improvement of participants after sequential attempts. More robust studies should administer standardized testing pre- and post- intervention to identify changes in ADHD control, such as through the ADHD Rating Scale-IV Test (49). Additionally, none of the studies compared their findings with a relevant control group, such as those without intervention or on pharmacological therapy, preventing an objective review of gamified digital interventions. Improvements in these aspects of study design will enable the scientific community to properly evaluate the cost-effectiveness of these games compared to current therapies and build a stronger case for game-based interventions to be a mainstay treatment for patients with ADHD.

### Patient Demographic

Bias observed in the demographics of patients studied suggests that the data collected might not be representative of the entire spectrum of patients with ADHD. All but two studies (15, 26) included only participants below the age of 16, despite ADHD being a frequently disabling disease in adulthood as well (50). Therapeutic options for adults with ADHD should not be trivialized. Studies can investigate the efficacy of these games for adults, accounting for differences in neuroplasticity of children's brains compared to matured brains and additional psychosocial considerations of adults with ADHD. Most studies were conducted in high-income countries, despite lower income countries being disproportionately affected by mental health disorders (51). Conducting research in lower income countries allow us to evaluate game-based interventions under separate circumstances, where barriers to access and differing attitudes toward ADHD may influence treatment efficacy. Future studies can also investigate if game-based interventions are more efficacious in specific subsets of the patient population. While briefly explored in Spitale et al. (28), which optimized games for milder vis-à-vis severe ADHD, more can be done to establish different indications for varying presentations of ADHD symptoms. Improvements in the study of patient demographics translates into target care, generating better treatment outcomes.

## Conclusion

There is an abundance of mobile game applications available for the diagnosis, management, and education of ADHD patients and their caregivers. The integration of these games with existing treatment options represents an opportunity to extend the management of ADHD beyond geographical boundaries, promote greater self-responsibility, and allow patients to receive treatment at the convenience of their own homes, at any time of the day. Optimizing the applications by reflective evidence from the stake holders is essential, as the evidence could be helpful in developing a robust evidence-based prototype. It is advantageous to use commercially available game applications pertaining their smoother and appealing appearance, however, the drawbacks of these apps should be set before use.

Most of the reviewed games had not undergone stringent clinical testing and thus while well-perceived by ADHD patients, their clinical utility remains questioned. Moving forwards, the conduct of some robust clinical trials examining the effectiveness of these applications would help to promote their use further.

## Data Availability Statement

The original contributions presented in the study are included in the article/Supplementary Material, further inquiries can be directed to the corresponding author.

## Author Contributions

MZ and RV jointly conceptualized the study and provided critical inputs to the final manuscript. HJ, RN, YS, and LR were involved in the identification of relevant articles and writing the initial draft, which was revised by HJ, RN, YS, LR, and RV. All authors have read and approved the manuscript prior to submission.

## Conflict of Interest

The authors declare that the research was conducted in the absence of any commercial or financial relationships that could be construed as a potential conflict of interest.

## Publisher's Note

All claims expressed in this article are solely those of the authors and do not necessarily represent those of their affiliated organizations, or those of the publisher, the editors and the reviewers. Any product that may be evaluated in this article, or claim that may be made by its manufacturer, is not guaranteed or endorsed by the publisher.
