# Supplementary material for: The Use of Mobile Games in the Management of Patients With Attention Deficit Hyperactive Disorder: A Scoping Review
Source: Front Psychiatry. 2022 Mar 4;13:792402. doi: 10.3389/fpsyt.2022.792402 (PMC8931195; doi:10.3389/fpsyt.2022.792402)
Supplement: Supplementary file 1 [file Data_Sheet_1.docx]

| **Feature** | **MeSH term** | **Synonyms** | **Others** |
| --- | --- | --- | --- |
| Games | Play and Playthings   - Games, Recreational - Video Games | Computer Games  Mobile Games | Serious game*  Serious gamingm  Video-gam*  Mobile gam*  Mobile-gam* |
| Mobile/tablet | Cell Phone Use  Telemedicine   - Telerehabilitation   Mobile Applications  Computers, handheld | Mobile Phone Use  Mobile Apps  Portable Electronic Applications  Portable Electronic Apps  Portable Software Applications  Portable Software Apps | m-learn*  mlearn*  smartphone*  smart-phone*  mobile-phone*  cell-phone*  Iphone*  Android*  Tablet*  Ipad*  Handheld computer* |
| ADHD | Attention Deficit Disorder with Hyperactivity | ADHD  ADDH  Attention Deficit Disorder  Attention Deficit Disorders with Hyperactivity  Attention Deficit Hyperactivity Disorder  Attention Deficit Hyperactivity Disorders  Attention Deficit-Hyperactivity Disorder  Brain Dysfunction, Minimal  Hyperkinetic Syndrome  Minimal Brain Dysfunction |  |

**Supplementary Appendix S1**

**MEDLINE (Ovid)**

1. exp Games, Recreational/
2. exp Video Games/
3. (mobile gam* or mobile-gam* or computer gam* or video-gam* or serious gam* or serious gaming* or game or gaming or play or recreation* or digital media*).mp. [mp=title, abstract, original title, name of substance word, subject heading word, floating sub-heading word, keyword heading word, organism supplementary concept word, protocol supplementary concept word, rare disease supplementary concept word, unique identifier, synonyms]
4. 1 or 2 or 3
5. exp Cell Phone/
6. exp Telemedicine/
7. exp Mobile Applications/
8. exp Computers, Handheld/
9. (Mobile phone* or mobile app* or portable electronic app* or portable software app* or m-learn* or mlearn* or smartphone* or smart-phone* or mobile-phone* or mobile* or cell phone* or cell-phone* or Iphone* or android* or tablet* or handheld computer* or app or application*).mp. [mp=title, abstract, original title, name of substance word, subject heading word, floating sub-heading word, keyword heading word, organism supplementary concept word, protocol supplementary concept word, rare disease supplementary concept word, unique identifier, synonyms]
10. 5 or 6 or 7 or 8 or 9
11. exp Attention Deficit Disorder with Hyperactivity/
12. (ADHD or ADDH or Attention Deficit Disorder* or Hyperactivity Disorder Attention Deficit or Hyperactivity Disorder* or Attention Deficit-Hyperactivity Disorder* or Brain Dysfunction, Minimal or Hyperkinetic Syndrome or Minimal Brain Dysfunction*).mp. [mp=title, abstract, original title, name of substance word, subject heading word, floating sub-heading word, keyword heading word, organism supplementary concept word, protocol supplementary concept word, rare disease supplementary concept word, unique identifier, synonyms]
13. 11 or 12
14. 4 and 10 and 13
15. limit 14 to yr="2010 -Current"
16. limit 15 to english language

(31 results)

**Cochrane**

1. MeSH descriptor: [Games, Recreational] explode all trees
2. MeSH descriptor: [Video Games] explode all trees
3. mobile gam* or mobile-gam* or computer gam* or video-gam* or serious gam* or serious gaming* or game or gaming or play or recreation* or digital media*
4. #1 or #2 or #3
5. MeSH descriptor: [Cell Phone] explode all trees
6. MeSH descriptor: [Telemedicine] explode all trees
7. MeSH descriptor: [Mobile Applications] explode all trees
8. MeSH descriptor: [Computers, Handheld] explode all trees
9. Mobile phone* or mobile app* or portable electronic app* or portable software app* or m-learn* or mlearn* or smartphone* or smart-phone* or mobile-phone* or mobile* or cell phone* or cell-phone* or Iphone* or android* or tablet* or handheld computer* or app or application*
10. #5 or #6 or #7 or #8 or #9
11. MeSH descriptor: [Attention Deficit Disorder with Hyperactivity] explode all trees
12. ADHD or ADDH or Attention Deficit Disorder* or Hyperactivity Disorder Attention Deficit or Hyperactivity Disorder* or Attention Deficit-Hyperactivity Disorder* or Brain Dysfunction, Minimal or Hyperkinetic Syndrome or Minimal Brain Dysfunction*
13. #11 or #12
14. #4 and #10 and #13 (with Cochrane Library publication date from Jan 2010 to Jul 2021)

(354 results)

**PsychInfo (EBSCO)**

S1: MA game, recreational OR MA video games OR ( mobile gam* or mobile-gam* or computer gam* or video-gam* or serious gam* or serious gaming* or game or gaming or play or recreation* or digital media* )

S2: MA cell phones OR MA telemedicine OR MA mobile applications OR MA computers, handheld OR ( Mobile phone* or mobile app* or portable electronic app* or portable software app* or m-learn* or mlearn* or smartphone* or smart-phone* or mobile-phone* or mobile* or cell phone* or cell-phone* or Iphone* or android* or tablet* or handheld computer* or app or application* )

S3: MA Attention Deficit Disorder with Hyperactivity OR ( ADHD or ADDH or Attention Deficit Disorder* or Hyperactivity Disorder Attention Deficit or Hyperactivity Disorder* or Attention Deficit-Hyperactivity Disorder* or Brain Dysfunction, Minimal or Hyperkinetic Syndrome or Minimal Brain Dysfunction* )

S4: S1 AND S2 AND S3

S5: S1 AND S2 AND S3 (Limiters - Publication Year: 2010-2021)

S6: S1 AND S2 AND S3 (Narrow by Language: - English)

(77 results)

**Scopus**

TITLE-ABS-KEY("Games, Recreational" OR "Video Games" OR "mobile gam*" OR "mobile-gam*" OR "computer gam*" OR "video-gam*" OR "serious gam*" OR "serious gaming*" OR "game" OR "gaming" OR "play" OR "recreation*" OR "digital media*") AND TITLE-ABS-KEY("Cell Phone" OR "Telemedicine" OR "Mobile Applications" OR "Computers, Handheld" OR "Mobile phone*" OR "mobile app*" OR "portable electronic app*" OR "portable software app*" OR "m-learn*" OR "mlearn*" OR "smartphone*" OR "smart-phone*" OR "mobile-phone*" OR "mobile*" OR "cell phone*" OR "cell-phone*" OR "Iphone*" OR "android*" OR  "tablet*" OR "handheld computer*" OR "app" OR "application*") AND TITLE-ABS-KEY("Attention Deficit Disorder with Hyperactivity" OR "ADHD" OR "ADDH" OR "Attention Deficit Disorder*" OR "Hyperactivity Disorder Attention Deficit" OR "Hyperactivity Disorder*" OR "Attention Deficit-Hyperactivity Disorder*" OR "Brain Dysfunction, Minimal" OR "Hyperkinetic Syndrome" OR "Minimal Brain Dysfunction*") AND ( LIMIT-TO ( PUBYEAR , 2021 ) OR LIMIT-TO ( PUBYEAR , 2020 ) OR LIMIT-TO ( PUBYEAR , 2019 ) OR LIMIT-TO ( PUBYEAR , 2018 ) OR LIMIT-TO ( PUBYEAR , 2017 ) OR LIMIT-TO ( PUBYEAR , 2016 ) OR LIMIT-TO ( PUBYEAR , 2015 ) OR LIMIT-TO ( PUBYEAR , 2014 ) OR LIMIT-TO ( PUBYEAR , 2013 ) OR LIMIT-TO ( PUBYEAR , 2012 ) OR LIMIT-TO ( PUBYEAR , 2011 ) OR LIMIT-TO ( PUBYEAR , 2010 ) ) AND ( LIMIT-TO ( LANGUAGE , "English" ) )

(199 results)
